# Prognostic value of prognostic nutritional index in patients with heart failure: a systematic review and meta-analysis

**DOI:** 10.3389/fcvm.2026.1751210

**Published:** 2026-06-19

**Authors:** Keyi Dong, Cailiang Peng, Zichao Song, Naizhi Geng

**Affiliations:** 1 First Clinical Medical College, Heilongjiang University of Chinese Medicine, Harbin Heilongjiang, China; 2 Department of Cardiovascular Medicine III, The First Affiliated Hospital of Heilongjiang University of Chinese Medicine, Harbin Heilongjiang, China

**Keywords:** all-cause mortality, cardiovascular mortality, heart failure, hospitalization rate for first heart failure, prognosis, prognostic nutritional index

## Abstract

**Background:**

According to the latest research data, the prognostic nutritional index (PNI) is closely associated with the prognosis of patients suffering from heart failure (HF). However, there remains controversy regarding the findings of existing studies.

**Methods:**

A systematic search was conducted up to March 27, 2025, across multiple databases, including PubMed, Embase, Web of Science, and the Cochrane Library. Observational studies assessing the prognostic value of PNI in patients with HF were identified. The main outcomes included all-cause mortality, cardiovascular mortality, and the rate of first hospitalization for HF. Among these outcomes, all-cause mortality and cardiovascular mortality were analyzed using hazard ratios (HRs) and 95% confidence intervals (95% CIs), while the rate of first hospitalization for HF was evaluated using odds ratios (ORs) and 95% CIs. Additionally, sensitivity and subgroup analyses were conducted to assess the robustness of the results and explore potential sources of heterogeneity.

**Results:**

A total of 14 cohort studies were included, involving 14,166 patients with HF. The pooled data showed that, compared with HF patients with lower PNI, those with higher PNI had lower risks of all-cause mortality (HR = 0.72, 95% CI: 0.66–0.79; *p* < 0.00001), cardiovascular mortality (HR = 0.77, 95% CI: 0.67–0.89; *p* = 0.0003), and first hospitalization for HF (OR = 0.73, 95% CI: 0.65–0.82; *p* < 0.00001).

**Conclusion:**

The available evidence suggests that PNI can be used for prognostic assessment in patients with HF and has the potential to serve as a prognostic biomarker. PNI was significantly inversely associated with adverse outcomes in patients with HF; that is, lower PNI values were associated with poorer prognosis.

**Systematic Review Registration:**

https://www.crd.york.ac.uk/PROSPERO/home.

## 1 Introduction

Heart failure (HF) stands as a leading contributor to global morbidity and mortality. According to relevant epidemiological data, the estimated number of people living with HF worldwide in 2017 was 64.3 million, and the overall incidence of HF continues to increase as the population ages (1). Although there have been advancements in the diagnosis and treatment of HF in recent years, the long-term prognosis of patients remains unsatisfactory. Currently, there is a lack of precise and effective prognostic assessment tools, which to some extent limits the formulation of individualized treatment plans and the rational allocation of medical resources. Therefore, further research into HF prognosis is of great importance for improving long-term patient outcomes.

Patients with HF generally exhibit poor nutritional status. By improving nutritional status, patients can experience enhanced cardiac function, improved quality of life, and reduced psychological stress. This intervention has considerable potential for clinical application (2). Buzby et al. first proposed the PNI in 1980 (3). The calculation is mainly based on two parameters: serum albumin and peripheral lymphocyte count. The calculation formula is: PNI(%) = 10 × Serum Albumin (g/L) + 0.005 × Total Lymphocyte Count (per mm^3^). This index provides a comprehensive assessment of nutritional and immune status (4). Fan et al. conducted a study in 2024 that included 218 elderly patients with HFpEF. After a follow-up of 59.02 ± 1.79 months, they found that PNI was significantly associated with all-cause mortality in elderly patients with HFpEF (5). In another study, Chien et al. investigated the prognostic value of PNI in patients with HFpEF in 2019. This study included 1,120 patients and demonstrated that PNI was significantly associated with both all-cause and cardiovascular mortality (6).

In 2022, Chen et al. conducted a similar meta-analysis (7). They searched four major databases—PubMed, Embase, Cochrane Library, and Google Scholar—for relevant observational studies published up to January 31, 2022. Ultimately, 14 eligible studies were included, involving a total of 19,605 HF patients with a median follow-up duration of 18.5 months. The study population encompassed HF subtypes including AHF, CHF, HFrEF, and HFpEF. Study quality was assessed using the Newcastle–Ottawa Scale, and hazard ratios (HRs) with 95% confidence intervals (CIs) were pooled using random-effects or fixed-effects models. The results showed that, in the multivariable-adjusted model, patients with low PNI had a 53% higher risk of all-cause mortality (HR = 1.53, 95% CI: 1.27–1.85) and a 126% higher risk of major adverse cardiovascular events (MACEs) (HR = 2.26, 95% CI: 1.54–3.31). For each 1-unit increase in PNI, the risks of all-cause mortality and MACEs decreased by 6% and 3%, respectively. Sensitivity analyses confirmed the robustness of the results. Moderate to high heterogeneity was observed, with *I*^2^ values of 71% and 64% for the analyses of all-cause mortality and MACEs, respectively. However, this meta-analysis had several important limitations, including the relatively early search date, the exclusion of outcomes related to first hospitalization for HF, and substantial heterogeneity in the analyses of the primary outcomes. These limitations further justify the need for the present updated systematic review and meta-analysis. Since the publication of that study, new clinical evidence has continued to emerge; however, the findings across studies remain inconsistent. These discrepancies may be attributable to differences in study populations, the definitions of outcome measures, and the PNI cut-off values used. Therefore, an updated meta-analysis is warranted to incorporate the most recent evidence and provide more comprehensive and reliable evidence to inform clinical practice.

## 2 Materials and methods

### 2.1 Literature search

This study was conducted in accordance with the Preferred Reporting Items for Systematic Reviews and Meta-Analyses (PRISMA 2020) statement (8). After finalization of the study protocol and prior to the commencement of the literature search, study screening, data extraction, and statistical analysis, this study was prospectively registered in the International Prospective Register of Systematic Reviews (PROSPERO: CRD420251057712). No material amendments were made to the registered protocol during the conduct of the study, ensuring the transparency and traceability of the research. The search strategy was developed by three investigators: DKY, PCL and SZC. They independently developed the subject terms and keywords and performed searches in PubMed, Embase, Web of Science, and the Cochrane Library. The search period covered the time from database inception to March 27, 2025. A broad range of search terms was used, including “Heart Failure,” “Cardiac Failure,” “Heart Decompensation,” “Congestive Heart Failure,” “Right-Sided Heart Failure,” “Left-Sided Heart Failure,” “Myocardial Failure,” “Prognostic Nutritional Index,” and “PNI.” The detailed search strategy is provided in Table 1.

**Table 1:** Detailed search strategy in four databases.

Database	Search strategy
Pubmed	((“Heart Failure”[Mesh]) OR ((((((((Cardiac Failure) OR (Heart Decompensation)) OR (Congestive Heart Failure)) OR (Right-Sided Heart Failure)) OR (Right Sided Heart Failure)) OR (Left-Sided Heart Failure)) OR (Left Sided Heart Failure)) OR (Myocardial Failure))) AND ((prognostic nutritional index[Title/Abstract]) OR (PNI))
Embase	((Heart Failure or (Cardiac Failure or Heart Decompensation or Congestive Heart Failure or Right-Sided Heart Failure or Right Sided Heart Failure or Left-Sided Heart Failure or Left Sided Heart Failure or Myocardial Failure)) and (prognostic nutritional index or PNI)).af.
Web of Science	((Heart Failure) OR ((((((((Cardiac Failure) OR (Heart Decompensation)) OR (Congestive Heart Failure)) OR (Right-Sided Heart Failure)) OR (Right Sided Heart Failure)) OR (Left-Sided Heart Failure)) OR (Left Sided Heart Failure)) OR (Myocardial Failure))) AND ((prognostic nutritional index) OR (PNI)) (Topic)
Chochrane	((Heart Failure or (Cardiac Failure or Heart Decompensation or Congestive Heart Failure or Right-Sided Heart Failure or Right Sided Heart Failure or Left-Sided Heart Failure or Left Sided Heart Failure or Myocardial Failure)) and (prognostic nutritional index or PNI)).af.

### 2.2 Study selection criteria

The inclusion criteria for this study were as follows: 1) Patients diagnosed with HF based on a standardized clinical assessment; 2) Studies that clearly defined PNI as the exposure variable and investigated its association with all-cause mortality, cardiovascular mortality, and first hospitalization for HF (defined as the first unplanned hospitalization due to worsening HF or newly developed HF symptoms); 3) Studies reporting odds ratios (ORs) or hazard ratios (HRs) with corresponding 95% confidence intervals (CIs), or providing sufficient data for their calculation; 4) Studies in which patients were categorized into high-PNI and low-PNI groups according to a predefined PNI cut-off value; and 5) Only studies published in full-text form were included. The exclusion criteria were as follows: 1) Review articles, expert commentaries, conference abstracts, case reports, and letters; 2) Studies lacking sufficient data to calculate ORs or HRs with 95% CIs; 3) Studies that did not report survival outcomes; and 4) Studies with duplicate or overlapping datasets.

### 2.3 Data extraction

Researchers DKY, PCL, and SZC independently extracted the data. Any discrepancies were resolved through discussion until consensus was reached among all authors. The extracted data included the first author's name, year of publication, study duration, country of origin, study design, HF classification, sample size, mean patient age, mean body mass index (BMI), PNI cut-off value, and multivariable-adjusted ORs or HRs (95% CIs) for PNI.

### 2.4 Study quality assessment

This study employed the Newcastle-Ottawa Scale (NOS) to assess the quality of the included studies. The maximum possible score is 9 points. Studies with scores ranging from 7 to 9 were considered high quality (9).

### 2.5 Statistical analysis

By pooling the multivariable-adjusted ORs or HRs and their corresponding 95% CIs (adjusted for age, sex, NYHA functional class, renal function, NT-proBNP, and other covariates), the prognostic value of PNI in patients with HF was systematically evaluated. Heterogeneity was assessed using Cochran's *Q* test and the *I*^2^ statistic, with significant heterogeneity defined as *P* < 0.1 or *I*^2^ > 50% (10). All data were pooled using a random-effects model. Furthermore, sensitivity and subgroup analyses were conducted to assess the robustness of the results and identify potential sources of heterogeneity. Publication bias was evaluated using funnel plots and Egger's test. A *P* value < 0.05 was considered statistically significant in all analyses. For outcomes with evidence of publication bias, the trim-and-fill method was applied to assess its potential impact on the results. Statistical analyses were performed using STATA 15.0 and Review Manager 5.4.

## 3 Results

### 3.1 Study characteristics

**Figure 1:**
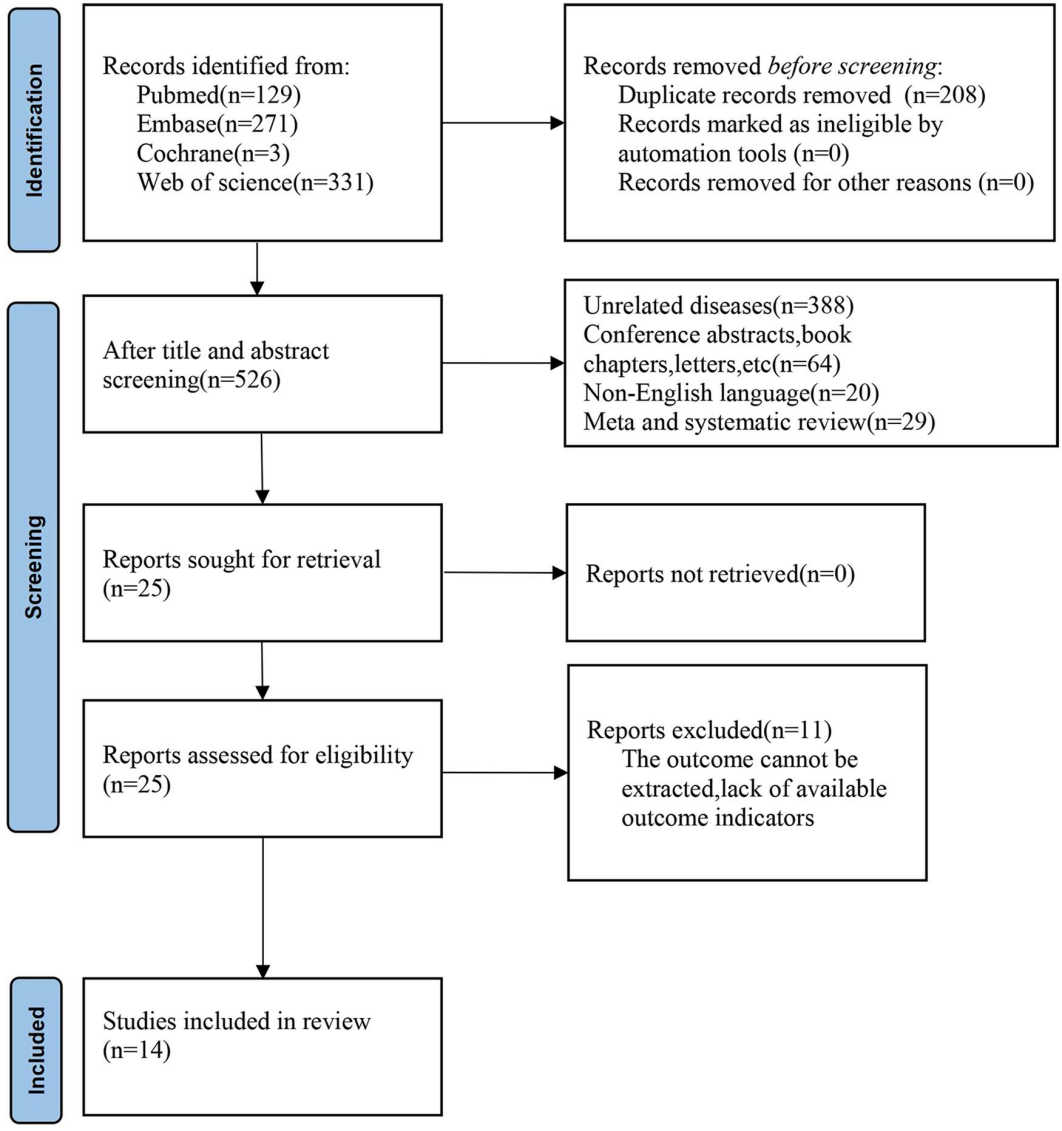
Flow chart of literature screening.

Using the predefined search strategy, a total of 734 records were identified through the initial database search. Of these, 208 records were removed as duplicates. After screening the titles and abstracts of the remaining records, 501 studies were excluded. Subsequently, 25 full-text articles were assessed for eligibility. Of these, 11 studies were excluded because of insufficient relevant data. Ultimately, 14 studies were included, involving a total of 14,166 patients (5, 6, 11–22) (Figure 1).

Among the 14 studies that met the inclusion criteria, 1 was conducted in the UK, 1 in Italy, 2 in Japan, 3 in Turkey, and 7 in China. Regarding study design, 12 were retrospective cohort studies (5, 6, 11, 13–20, 22), and 2 were prospective cohort studies (12, 21). Notably, the majority of the included studies adopted a retrospective cohort design, which may have introduced unmeasured selection bias and residual confounding. This methodological limitation should be considered when interpreting the pooled results. All included studies were published in English. Table 2 summarizes the characteristics of the study participants. Across the included studies, sample sizes ranged from 25 to 9,216 patients. The PNI cut-off values ranged from 34 to 63.25. Mean BMI ranged from 22.32 to 30.4 kg/m^2^, and mean age ranged from 46.7 to 85 years. The included populations comprised patients with HF, AHF, HFpEF, HFrEF, acute CHF exacerbations, acute decompensated HF, and metabolic syndrome with HF.

**Table 2:** Basic characteristics of the included literature.

Author	Study period	Region	Study design	Population	No. of patients	Gender		Mean/median age	Mean/median BMI	PNI cut-off
						Male	Female			
Akça (11)	2022−2022	Turkey	retrospective cohort	AHF	25	12	13	82	NA	35.6
Solano (12) a	NA	Britain	Prospective cohort	HFpEF	1101	517	584	73.5	30.3	48.5
Solano (12) b	NA	Britain	Prospective cohort	HFpEF	1265	615	650	72	30.4	51
Solano (12) c	NA	Britain	Prospective cohort	HFpEF	1045	520	525	70	30.1	54
Solano (12) d	NA	Britain	Prospective cohort	HFrEF	1944	1473	471	64.9	28.2	49.1
Solano (12) e	NA	Britain	Prospective cohort	HFrEF	1919	1503	416	63	28.3	51.95
Solano (12) f	NA	Britain	Prospective cohort	HFrEF	1942	1530	412	60	28.1	55.05
Fan (5) a	2016−2021	China	retrospective cohort	HFpEF	NA	NA	NA	85	NA	43.75
Fan (5) b	2016−2021	China	retrospective cohort	HFpEF	NA	NA	NA	85	NA	48.4
Zhuang (13) a	2015−2020	China	retrospective cohort	an acute episode of CHF	35	19	16	78.49	NA	35
Zhuang (13) b	2015−2020	China	retrospective cohort	an acute episode of CHF	50	35	15	79.04	NA	38
Yoshihisa (14)	2009−2015	Japan	retrospective cohort	HF	1108	792	316	66.5	NA	38
Chien (6)	2012–2014	China	retrospective cohort	HFpEF	680	NA	NA	77.2	NA	38
Gu (15) a	2017–2021	China	retrospective cohort	acute decompensated heart failure	196	121	75	71.35	22.32	40.8
Gu (15) b	2017–2021	China	retrospective cohort	acute decompensated heart failure	203	134	69	66.69	23.34	44.1
Gu (15) c	2017–2021	China	retrospective cohort	acute decompensated heart failure	197	111	86	66.55	22.93	44.2
Turen (16)	2014–2020	Turkey	retrospective cohort	Advanced Heart Failure	89	79	10	46.7	27.6	50.5
Shirakabe (17) a	2000–2016	Japan	retrospective cohort	AHF	77	45	32	75	NA	35
Shirakabe 2018 (17) b	2000–2016	Japan	retrospective cohort	AHF	50	36	14	79	NA	38
Zhang (18) a	2015–2019	China	retrospective cohort	Metabolic Syndrome and Heart Failure	292	197	95	67	26.71	45
Zhang (18) b	2015–2019	China	retrospective cohort	Metabolic Syndrome and Heart Failure	221	169	52	66	26.17	40
Cheng (19)	2003–2012	China	retrospective cohort	AHF	558	383	175	73.4	26.2	44.8
Çinier (20) a	2009–2019	Turkey	retrospective cohort	HFrEF	275	211	64	59	NA	52.1
Çinier (20) b	2009–2019	Turkey	retrospective cohort	HFrEF	275	221	54	63	NA	47.7
Çinier (20) c	2009–2019	Turkey	retrospective cohort	HFrEF	275	223	52	64	NA	40.1
Candeloro (21)	2016–2018	Italy	Prospective cohort	AHF	344	158	186	83.56	NA	34
Zhao (22) a	2008–2018	China	retrospective cohort	HF	NA	NA	NA	56.64	NA	43.6
Zhao (22) b	2008–2018	China	retrospective cohort	HF	NA	NA	NA	56.64	NA	48.55
Zhao (22) c	2008–2018	China	retrospective cohort	HF	NA	NA	NA	56.64	NA	63.25

NA, not available.

### 3.2 Study quality

Among the 14 studies, the NOS scores ranged from 5 to 8 (Table 3).

**Table 3:** Risk analysis of bias of included studies. (a) Quality evaluation of the eligible cohort studies with Newcastle-Ottawa scale; (a) Quality evaluation of the eligible cohort studies with Newcastle-Ottawa scale.

Study	Selection				Comparability		Outcome		
	Representative-ness	Selection of non-exposed	Ascertainment of exposure	Outcome not present at start	Comparability on most important factors	Comparability on other risk factors	Assessment of outcome	Long enough follow-up (median≥6 months)	Adequacy (completeness) of follow-up
Akça (11)	–	*	*	*	*	–	*	–	*
Solano (12)	–	*	*	*	–	–	*	*	*
Zhuang (13)	–	*	*	*	–	–	*	*	*
Yoshihisa (14)	*	*	*	*	–	–	*	*	*
Chien (6)	–	*	*	*	–	–	*	*	*
Gu (15)	*	*	*	*	–	–	*	–	*
Turen (16)	–	*	*	*	*	–	*	–	*
Shirakabe (17)	*	*	*	*	–	–	*	–	*
Zhang (18)	*	*	*	*	–	–	*	–	*
Cheng (19)	*	*	*	*	–	–	*	*	*
Candeloro (21)	*	*	*	*	*	–	*	*	*
Çinier (20)	–	*	*	*	–	*	*	*	*
Fan (5)	*	*	*	*	–	–	*	*	*
Zhao (22)	*	*	*	*	*	–	*	*	*

*indicates criterion met; – indicates significant of criterion not met.

### 3.3 Statistical results

#### 3.3.1 PNI and all-cause mortality

**Figure 2:**
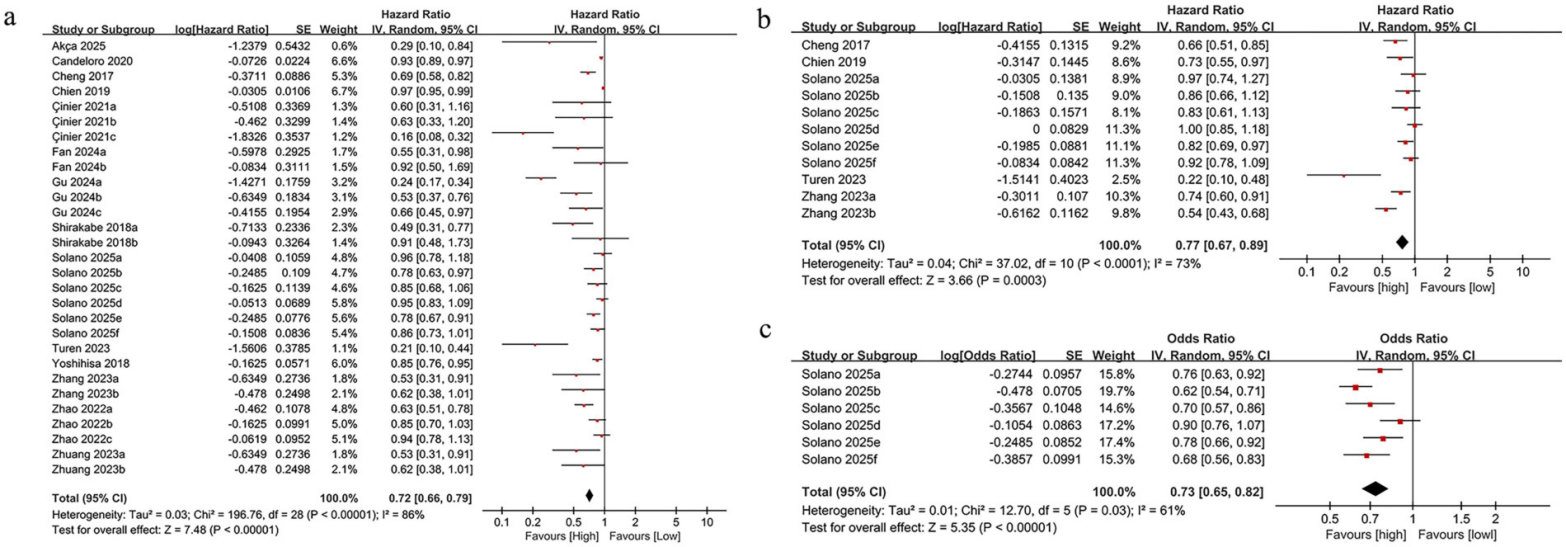
PNI was associated with All-cause mortality **(a)**, cardiovascular death **(b)** and first HF hospitalization **(c)**.

The association between PNI and all-cause mortality was investigated in 29 comparative groups, comprising a total of 14,166 participants. Given the substantial heterogeneity among the included studies (*I*^2^ = 86%, *p* < 0.00001, Figure 2a), a random-effects model was applied. The pooled analysis showed that higher PNI was significantly associated with a lower risk of all-cause mortality (HR = 0.72, 95% CI: 0.66–0.79; *p* < 0.00001, Figure 2a).

Subgroup analyses were performed to explore potential sources of heterogeneity in the association between PNI and all-cause mortality. The analyses were stratified according to HF subtype, patient age, geographic region, and PNI cut-off value. Notably, no statistically significant association between PNI and all-cause mortality was observed in the three studies conducted in Japan (HR = 0.73, 95% CI:0.51–1.06; *p* = 0.1, Table 4). Furthermore, between-study heterogeneity was substantially reduced after stratification by HF subtype and geographic region, suggesting that these factors may represent important sources of heterogeneity among the included studies.

**Table 4:** Subgroup analysis.

Subgroup	All-cause mortality			
	Study	HR [95%CI]	*P* value	*I* ^2^
Total	29	0.72[0.66–0.79]	<0.00001	86%
Type of heart failure				
AHF	10	0.57[0.42–0.76]	<0.00001	90%
HFpEF	6	0.89 [0.8–1]	0.05	44%
HFrEF	6	0.7 [0.54–0.9]	0.005	82%
Stable heart failure	7	0.71[0.59–0.86]	0.0006	76%
Mean/median age				
≥ 70y	14	0.74[0.65–0.83]	<0.00001	88%
<70y	15	0.7 [0.61–0.8]	<0.00001	77%
Country				
China	14	0.64[0.53–0.78]	<0.00001	90%
Turkey	5	0.33 [0.18–0.6]	0.0002	68%
Japan	3	0.73[0.51–1.06]	0.1	63%
British	6	0.87 [0.8–0.93]	0.0002	10%
Italy	1	0.93[0.89–0.97]	0.001	
PNI cut-off				
≥ 45	13	0.83[0.75–0.91]	0.0002	53%
<45	16	0.64[0.56–0.72]	<0.00001	91%

#### 3.3.2 PNI and cardiovascular death

Eleven studies contributed data on the association between PNI and cardiovascular mortality. Consistent with the findings for all-cause mortality, higher PNI was significantly associated with a lower risk of cardiovascular death (HR = 0.77, 95% CI: 0.67–0.89; *p* ＝ 0.0003, Figure 2b). A high degree of heterogeneity was observed among the included studies (*I*^2^ = 73%, *p* < 0.0001, Figure 2b).

#### 3.3.3 PNI and first hospitalization for HF

The association between PNI and first hospitalization for HF was also evaluated. Due to the heterogeneity among studies (*I*^2^ = 61%, *p* ＝ 0.03, Figure 2c), a random-effects model was applied. The pooled analysis showed that higher PNI was significantly associated with a lower risk of first hospitalization for HF (OR = 0.73, 95% CI: 0.65–0.82; *p* < 0.00001, Figure 2c).

### 3.4 Sensitivity analysis

**Figure 3:**
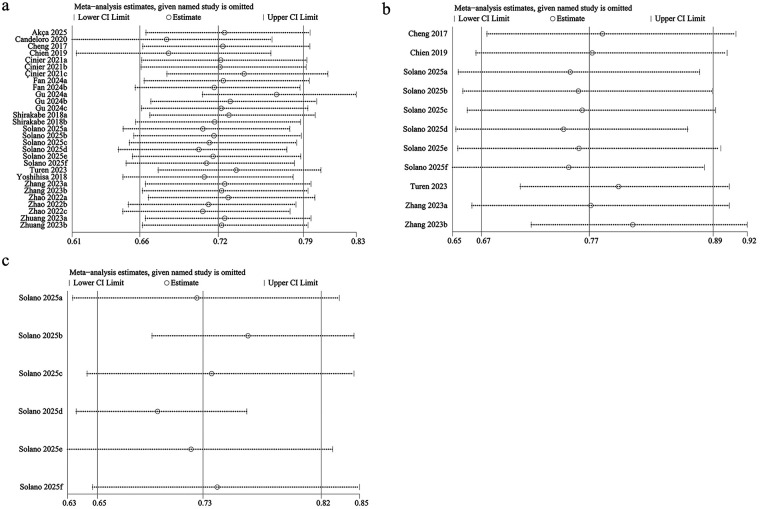
Sensitivity analysis of PNI with respect to **(a)** All-cause mortality **(b)** cardiovascular death and **(c)** first HF hospitalization.

To assess the robustness of the findings, sensitivity analyses were performed. The results showed that, after sequentially excluding each study, the pooled effect estimates remained within the 95% CI range of the original pooled estimate, indicating good robustness of the findings. Furthermore, no individual study had a disproportionate influence on the outcomes of all-cause mortality (Figure 3a), cardiovascular death (Figure 3b), or first hospitalization for HF (Figure 3c), supporting the reliability of the results.

### 3.5 Publication bias

**Figure 4:**
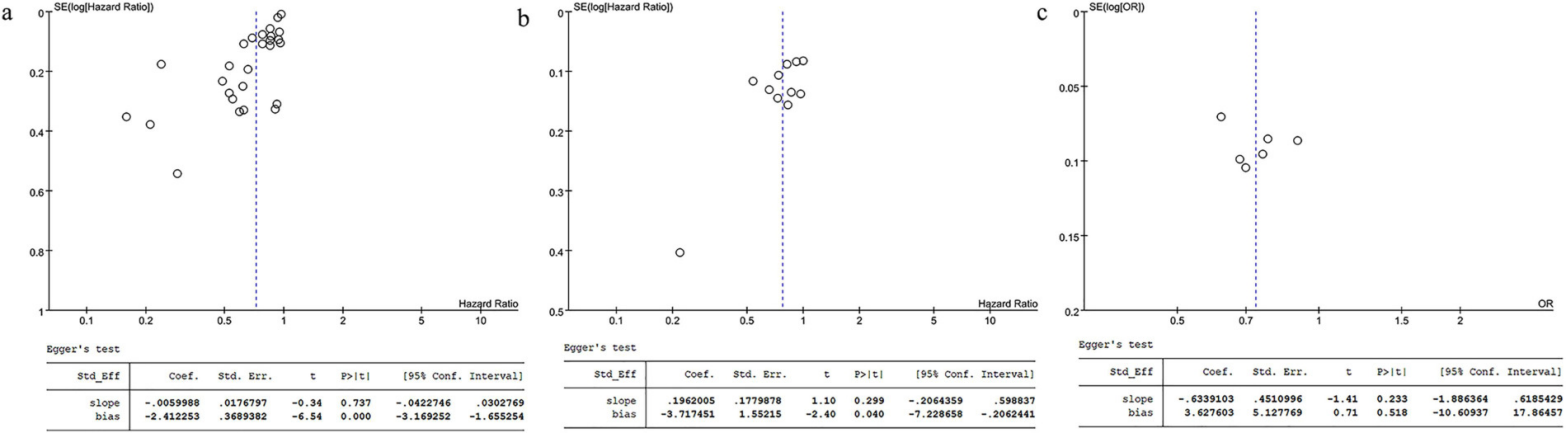
Funnel plot the evaluation of publication bias for **(a)** All-cause mortality and **(b)** cardiovascular death and **(c)** first HF hospitalization.

**Figure 5:**
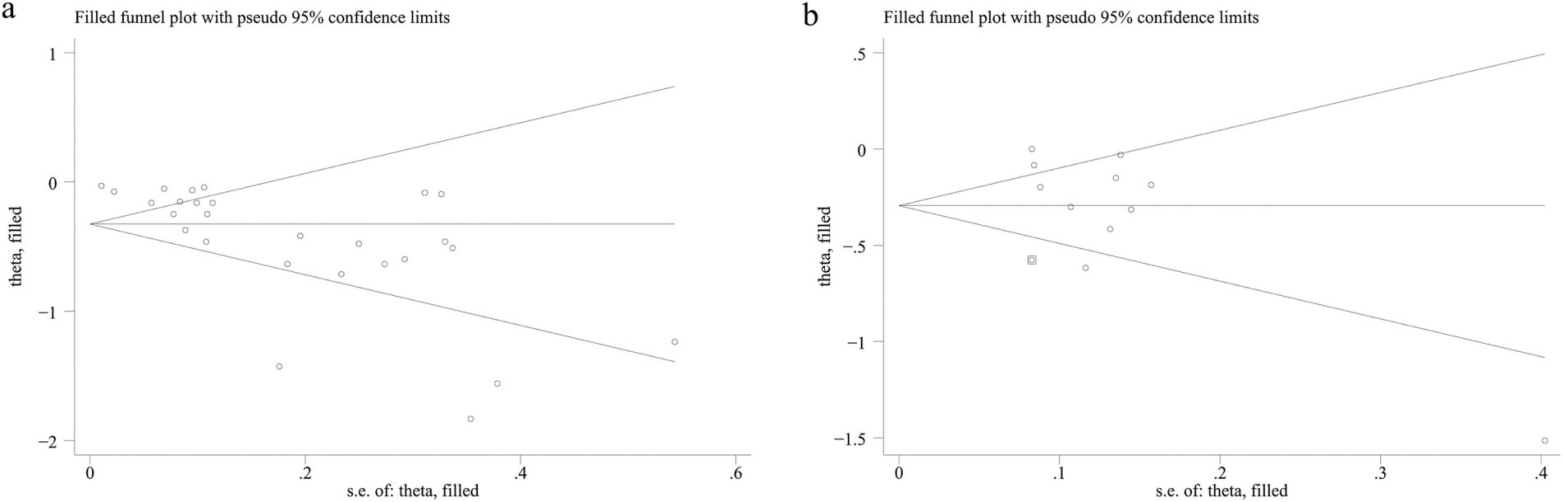
The funnel plot for **(a)** all-cause mortality and **(b)** cardiovascular mortality by the imputation method.

Publication bias was assessed for the three outcomes—all-cause mortality, cardiovascular mortality, and first hospitalization for HF—using funnel plots and Egger's test. The results indicated evidence of publication bias for the meta-analyses of all-cause mortality (Egger's test: *p* = 0.000) (Figure 4a) and cardiovascular death (Egger's test: *p* = 0.04) (Figure 4b). The impact of publication bias on all-cause mortality and cardiovascular death was evaluated using the imputation method. The results indicated that the significant association between the imputed PNI and all-cause mortality (OR = 0.72, 95% CI: 0.66–0.79) (Figure 5a) and cardiovascular death (OR = 0.75, 95% CI: 0.64–0.86) (Figure 5b) remained stable after imputation. Egger's test showed no significant publication bias in the meta-analysis of first hospitalization for HF (*p* = 0.518). Similarly, the funnel plot suggested no substantial publication bias for first hospitalization for HF (Figure 4c). In contrast, asymmetry was observed in the funnel plots for all-cause mortality (Figure 4a) and cardiovascular death (Figure 4b), further suggesting the presence of publication bias.

## 4 Discussion

Our study found that PNI was associated with all three outcomes: all-cause mortality, cardiovascular mortality, and first hospitalization for HF. Notably, the effect estimates included in the final pooled analyses were exclusively multivariable-adjusted ORs or HRs with their corresponding 95% CIs extracted from the original studies. The covariates adjusted for in these models generally included basic demographic characteristics such as age and sex, and some studies further adjusted for established prognostic factors for HF, including NYHA functional class, renal function, and NT-proBNP. Therefore, the pooled results of this study minimized the influence of known confounding factors as much as possible and provide preliminary evidence regarding the independent prognostic value of PNI for clinical outcomes in patients with HF. In addition, sensitivity analyses confirmed the stability and reliability of the effect estimates across all outcomes, indicating that the findings were not disproportionately influenced by any individual study.

However, we also noted substantial methodological heterogeneity across the included studies. There were marked differences in the covariates selected for adjustment in the multivariable regression models used by different studies. Furthermore, the prognostic value of PNI did not remain consistent after multivariable adjustment in all studies, and the statistical significance of PNI was attenuated or even lost in some studies following comprehensive adjustment for confounding factors. These findings suggest some uncertainty regarding the independent prognostic value of PNI in HF and warrant further investigation. In addition, evidence of publication bias was observed in the analyses of all-cause mortality and cardiovascular mortality, which may have affected the estimated effect sizes.

This study builds upon the work of Chen et al. in 2022 (7). It incorporates first hospitalization for HF as an additional outcome, further evaluating the prognostic value of PNI for this endpoint. In addition, comprehensive sensitivity and subgroup analyses were performed to assess the robustness of the findings. Potential sources of heterogeneity and populations in which these associations may be most relevant were also explored. Therefore, this study provides more comprehensive evidence than the previous meta-analysis.

To further investigate the association between PNI and all-cause mortality, subgroup analyses were performed. In the Japanese subgroup, no significant association was observed between PNI and all-cause mortality. There are two possible explanations for this finding. First, the limited number of studies conducted in Japan may have resulted in insufficient statistical power and a false-negative result. Second, this finding may reflect differences in HF mortality across countries and regions. In low-income and lower-middle-income countries, although hospitalization rates for HF are among the lowest worldwide, mortality rates are approximately twice those observed in high-income countries (23, 24).

In this study, funnel plots and Egger's test were used to assess publication bias for the three primary outcomes: all-cause mortality, cardiovascular mortality, and first hospitalization for HF. The results indicated evidence of publication bias for all-cause mortality and cardiovascular mortality, whereas no statistically significant publication bias was detected for first hospitalization for HF. One possible explanation for the observed publication bias is the well-recognized tendency toward positive publication bias in clinical research. Among small-sample studies evaluating the association between PNI and HF prognosis, studies reporting significant associations between low PNI and adverse clinical outcomes may be more likely to be submitted and published. In contrast, studies reporting non-significant findings may be less likely to be published. Consequently, the overall distribution of effect estimates among the included studies may be skewed toward positive findings, contributing to the observed publication bias. This bias may lead to an overestimation of the pooled effect size. Specifically, it may exaggerate the apparent protective association between higher PNI and the risks of all-cause mortality and cardiovascular mortality in patients with HF, resulting in a discrepancy between the pooled estimate and the true underlying effect.

To further quantify the impact of publication bias on the robustness of our core conclusions, the non-parametric trim-and-fill method was adopted to perform bias correction for the two outcomes with significant publication bias, namely all-cause mortality and cardiovascular mortality. The results showed that after supplementing and estimating the missing studies with negative results, the adjusted pooled effect size for all-cause mortality still maintained the same association direction as the original analysis. Specifically, higher PNI levels remained significantly associated with a lower risk of all-cause mortality in HF patients, with only a slight attenuation in the magnitude of the effect size compared with the original result. The 95% CI still did not cross the line of no effect, and the statistical significance did not essentially change. For cardiovascular mortality, the pooled effect size after trim-and-fill adjustment also retained the original direction of association, with only a minor reduction in the effect magnitude while still maintaining statistical significance. These results indicated that although a certain degree of publication bias existed for the two core outcomes in this study, and this bias may have exaggerated the apparent protective effect of PNI to a certain extent, the core conclusions of our study did not show a directional reversal after trim-and-fill correction. The findings continue to support the view that PNI may serve as a useful biomarker for prognostic evaluation in patients with HF, supporting the robustness of the main conclusions. These findings also suggest several directions for future research. First, international multicenter, large-sample prospective cohort studies should be conducted. A uniform set of covariates for multivariable adjustment should be predefined at the stage of study design, along with a standardized definition of the PNI cut-off value and adjudication criteria for endpoint events. This may help reduce methodological heterogeneity across studies and clarify the independent incremental prognostic value of PNI for the prognosis of patients with HF. Second, individual patient data (IPD) meta-analysis should be performed. By integrating individual-level data of original patients and adopting a unified adjustment model and statistical analysis strategy, this approach can eliminate the bias arising from inconsistent adjustment models between studies and obtain more robust effect estimates that are more representative of real-world clinical practice. Third, RCTs are needed to determine whether PNI-targeted nutritional interventions can improve the clinical prognosis of patients with HF, so as to provide high-level evidence for the clinical translation and application of PNI. Fourth, at present, there is no universally recognized and unified cut-off value definition standard for PNI, which limits the clinical applicability of this indicator and also makes it impossible to directly compare the results of various studies. In the future, large-sample studies should be conducted to determine and verify its optimal critical value.

The key finding of this study is that nutritional status is associated with the prognosis of patients with HF. Nutritional assessment of patients with HF and the identification of accurate assessment tools are fundamental for developing effective nutritional strategies and have a significant impact on patient treatment and management. PNI is a biomarker that reflects the nutritional status of patients with HF. Malnutrition contributes to HF progression by exacerbating protein metabolism disorders and excessive autophagic activation. By regulating the mTOR pathway and inhibiting excessive and harmful autophagy, nutritional intervention may become an important adjunctive strategy for improving the prognosis of patients with HF. Future research should further clarify the optimal nutritional strategies and their underlying molecular mechanisms (25). It is worth noting that the prognostic value of nutritional scores is not limited to HF. Recent studies have also shown that nutritional scores have predictive value for outcomes in patients with non-valvular atrial fibrillation (26), while metabolic indicators such as the triglyceride-glucose index are associated with the risk of complications following coronary intervention (27). These findings collectively highlight the potential importance of integrating systematic nutritional and metabolic assessments into comprehensive cardiovascular risk management. In addition, PNI is also associated with inflammatory markers. In a previous study, researchers reviewed the clinical data of 274 patients with stage III colon cancer who underwent radical surgery followed by adjuvant chemotherapy and analyzed the relationship between PNI and related indicators. The results showed that low PNI levels were significantly associated with markers of systemic inflammation in patients with stage III colon cancer. This finding supports the clinical value of PNI as an indicator reflecting the interaction between nutritional and inflammatory status (28).

Although this meta-analysis provides valuable information, several limitations should be acknowledged. First, most of the included studies were conducted in Asia, particularly in China. Second, the majority of the included studies adopted a retrospective rather than a prospective design, which may limit the overall level of evidence. Third, the PNI cut-off values used to define malnutrition varied across studies, which may represent an important source of heterogeneity. Fourth, the overall sample size included in the analysis was relatively small. Fifth, publication bias was observed in the meta-analyses of both all-cause mortality and cardiovascular mortality. Overall, whether interventions aimed at improving PNI can positively affect the prognosis of patients with HF requires further investigation.

## 5 Conclusion

The results of this meta-analysis indicate that PNI has potential prognostic value for all-cause mortality, cardiovascular mortality, and the first hospitalization for HF, and lower PNI levels may be associated with worse clinical outcomes in this population. Subgroup analyses suggested that geographic region may influence the prognostic performance of PNI in HF prognosis. However, constrained by several limitations of the included studies, including the predominantly retrospective study design, the majority of study populations being recruited from Asian regions, inconsistent multivariable adjustment models across studies, and the presence of publication bias for some endpoints, the independent incremental prognostic value of PNI in patients with HF remains to be further validated. Therefore, future international multicenter, large-sample prospective studies, as well as IPD meta-analyses adopting a unified adjustment model, are warranted to definitively clarify the optimal applicable population, optimal cut-off value, and clinical application value of PNI in the prognostic assessment of HF.

## Data Availability

The original contributions presented in the study are included in the article/Supplementary Material, further inquiries can be directed to the corresponding author.
